# Newspapers and Periodicals

**Published:** 1880-02

**Authors:** 


### NEWSPAPERS AND PERIODICALS.

Whose mind does not run back to the sunnier days of childhood at the repetition of the dear familiar lines:

> "In works of labor or of skill,
> I would be busy too;
> For Satan finds some mischief still
> For idle hands to do."

And how much better, too, is the good old Presbyterian custom of causing children to commit to memory such plain and wholesome truths, than of lumbering up their brains with the doggerel rhymes of *Old Mother Goose*, and such as

> "There was an old woman, she lived in a spoon,

> And all she wanted was elbow room."

Then again:

> "O, Miss Mary, quite contrary,
> How doos your garden grow?"
> "Silver bells and muscle shells,
> And cucumbers all in a row."

Eminently suggestive are such lines of—nonsense.

But what connection is there between newspapers and idle children? There is no connection whatever, reader, and that is precisely the point we are trying to make. We wish to express it as a mature conviction of our own mind, that one of the best protections for our children against the temptations of city and village life, is the habitual reading of a well-conducted family newspaper or periodical. If you want a child to take an interest in a paper, let it be *his* paper, sent to his address. In a reasonable time he will get to look for its coming, and feel the want of it, if it does not arrive at the usual time. Soon it will be a kind of necessity, and rather than be without it he becomes willing to make sacrifices and self-denials for the sake of saving any stray dime or half-dime which may happen to come into his possession. Peanuts and gingerbread, monkey-shows and fire-crackers, are vetoed, and the increment of a quarter of a dollar to a half, and so on, to the subscription price, is watched with an interest and a pleasure which few would imagine, and lo! the germs of an economy and a self-denial are planted before we are aware of it, which will grow to health, and wealth, and position.

